# Dual Resonator MEMS Magnetic Field Gradiometer

**DOI:** 10.3390/s19030493

**Published:** 2019-01-25

**Authors:** Matthias Kahr, Michael Stifter, Harald Steiner, Wilfried Hortschitz, Gabor Kovács, Andreas Kainz, Johannes Schalko, Franz Keplinger

**Affiliations:** 1 Department for Integrated Sensor Systems, Danube University Krems, 2700 Wiener Neustadt, Austria; michael.stifter@donau-uni.ac.at (M.S.); harald.steiner@donau-uni.ac.at (H.S.); wilfried.hortschitz@donau-uni.ac.at (W.H.); gabor.kovacs@donau-uni.ac.at (G.K.); 2 Institute of Sensor and Actuator Systems, TU Wien, 1040 Vienna, Austria; andreas.kainz@tuwien.ac.at (A.K.); johannes.schalko@tuwien.ac.at (J.S.); franz.keplinger@tuwien.ac.at (F.K.)

**Keywords:** MEMS, Gradiometer, Lorentz force, magnetic field, sensor, optical readout

## Abstract

Accurate knowledge of the spatial magnetic field distribution is necessary when measuring field gradients. Therefore, a MEMS magnetic field gradiometer is reported, consisting of two identical, but independent laterally oscillating masses on a single chip. The sensor is actuated by Lorentz force and read out by modulation of the light flux passing through stationary and moving arrays of the chip. This optical readout decouples the transducer from the electronic components. Both phase and intensity are recorded which reveals information about the uniformity of the magnetic field. The magnetic flux density is measured simultaneously at two points in space and the field gradient is evaluated locally. The sensor was characterised at ambient pressure by performing frequency and magnitude response measurements with coil and various different permanent magnet arrangements, resulting in a responsivity of 35.67 V/T and detection limit of 3.07 µT/$\sqrt{\mathrm{Hz}}$ (@ 83 Hz ENBW). The sensor is compact, offers a large dynamic measurement range and can be of low-cost by using conventional MEMS batch fabrication technology.

## 1. Introduction

The measurement of magnetic fields is key for many scientific and technical areas and, thus, many different approaches have been investigated and developed in the last decades, including NMR (nuclear magnetic resonance), SQUID (superconducting quantum interference device), Hall effect sensors, fluxmeters, sensors based on the MR (magneto resistive) effect and MEMS (micro-electro-mechanical system) resonant magnetometers. SQUID and NMR measurement techniques are highly accurate but rather cumbersome and expensive and usually deployed at the scientific community [1]. Additionally, a SQUID must be cooled with liquid helium to maintain superconductivity. Hall effect sensors and MR sensors are currently part of the dominant technology in consumer electronic devices. Nevertheless, these elements are limited in their measurement range and suffer from saturation effects [2]. Furthermore, Hall probes strongly influenced by temperature changes and geometrical induced non-linearities [3], whereas MR sensors show magnetic hysteresis due to their ferromagnetic thin-film elements [4].

In contrast, silicon MEMS magnetometers based on Lorentz force-excited resonators benefit from magnitude amplification and a large dynamic range [5,6]. For measuring time-invariant magnetic fields, these amplitude modulated sensors are excited at their natural frequency $f_{r}$ with an alternating current $i_{\mathrm{ac}}$. Thus, interfering signals with $f\neq f_{r}$ are suppressed and the motion induced by Lorentz force is increased by the sensor’s quality factor *Q*. This maximises the magnetic field sensitivity. Another approach to measure magnetic fields based on resonant MEMS sensors is to integrate magnetic particles onto a cantilever [7]. Small sizes and high magnetic field resolution are feasible. Unlike Lorentz force-excited resonators, the dynamic range is very limited due to the absence of an adjustable excitation current.

Table 1 compares various Lorentz force-based MEMS magnetic field sensors recently published in the literature.

Concerning gradient field measurements, different measurement techniques have been investigated in the recent years, such as the deployment of a single string as sensing element [12], a membrane with a fixed permanent magnet [13] or coupled sensing elements [14].

The reported magnetic field gradiometer features light flux modulation through static and deflectable gratings and an optical readout which decouples the sensing part from the electronic components. The optical readout is highly sensitive and was investigated in [15,16,17]. Recently, an optical Lorentz force transducer comprising a single mass for magnetic field detection was characterised and published in [18]. MEMS based gradient field sensors have their potential application in non-destructive testing [19], environmental mapping and navigation [20], magnetic dipole characterisation (e.g., at CERN [21]), MRI (Magnetic Resonance Imaging) worker monitoring [22] and many more.

## 2. Sensing Principle and Fabrication

**Figure 1:**
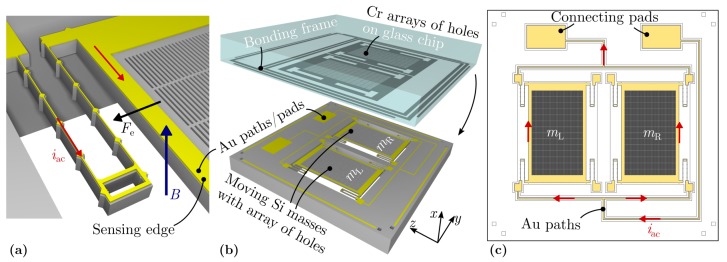
(**a**), ThreeD rendering of the sensor’s spring and illustration of the Lorentz force principle. (**b**), Exploded 3D-model of the sensor chip showing both masses, gold (Au) conducting paths and the stationary Cr pattern on glass. (**c**), Layout of gold paths and current distribution of the sensor.

A silicon (Si) microstructure was designed incorporating two identical movable masses with gratings and gold conductor paths (Figure 1a,b). The device was fabricated on wafer-level scale with silicon-on-insulator (SOI) technology. First, the gold conducting paths were patterned with lift-off technique. Subsequently, the microstructures comprising both masses were structured by photolithography and deep reactive ion etching (DRIE) of the device layer (45 µm) of a SOI wafer. A protective layer of photoresist was added to support the structures during the subsequent backside etch up to the intermediate SiO${}_{2}$ layer. Following this, the oxide layer was removed by wet chemical etching. A glass carrier was patterned with Chromium (Cr) gratings by physical vapor deposition (PVD). The carrier was then bonded to the SOI wafer which was subsequently diced into 6 mm × 6 mm chips with a wafer saw. Finally, the protective layer was removed to release the movable microstructures. In presence of a static magnetic field *B*, the masses are excited with Lorentz force $F_{e}=L_{e}\;i_{\mathrm{ac}}\;B$ and driven at resonance controlled with a sinusoidal current $i_{\mathrm{ac}}$. The current is fed in via the outermost springs alongside the edge of length $L_{e}=2.5\;\mathrm{mm}$ of the masses. This is, at the same time, the sensing edge which the force acts on. Due to the design, the sensing edges of both masses are separated by a fixed distance of $d_{\mathrm{sens}}=3.9\;\mathrm{mm}$. The single current feed is divided into two parallel paths before it accesses the masses and is rejoined afterwards (Figure 1c). Thus, only two connecting pads are necessary to guide $i_{\mathrm{ac}}$. The U-shaped springs (with a large height-to-width ratio) allow lateral deflections of the masses and prevents mechanical non-linearities.

**Figure 2:**
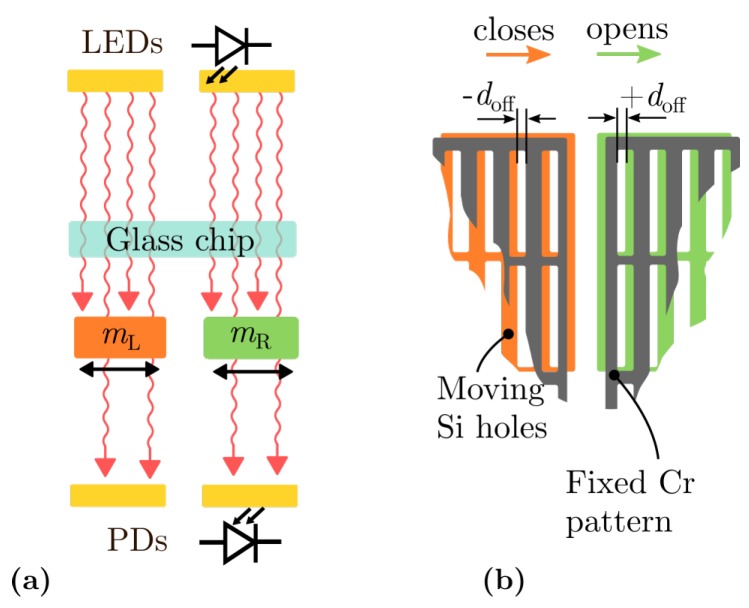
(**a**), Schematic of the modulation principle. The light flux emitted by LEDs is passing through the glass chip and the deflectable masses, and then detected by photodiodes. (**b**), Designed offset $d_{\mathrm{off}}$ between stationary Cr pattern and Si holes.

The readout of the Lorentz force induced deflection is achieved by observing the modulated light flux through the stationary Cr grid and movable Si grid. Two photodiodes, one below each mass, detects the change in the light flux yielding the light intensity and phase information corresponding to the magnetic field strength (Figure 2a). By design, the opening of the masses and grating of the glass mask are displaced in a way, thus guaranteeing the half net-light flux passing through the openings when the device is idle. Each grid consists of 1300 rectangular openings, with a width of 5 µm and length of 100 µm. In addition, the mask offset is $-d_{\mathrm{off}}$ and $+d_{\mathrm{off}}$ for the mass $m_{L}$ located on the left side and $m_{R}$ on the right side of the Si chip, respectively (Figure 2a). When both masses deflect in the same direction, one mass will close the apertures whereas the other will open them. From this, the direction of the magnetic field will be obtained by monitoring the phase signal of the detected light flux related to the reference signal ($i_{\mathrm{ac}}$).

### Transduction Scheme

**Figure 3:**
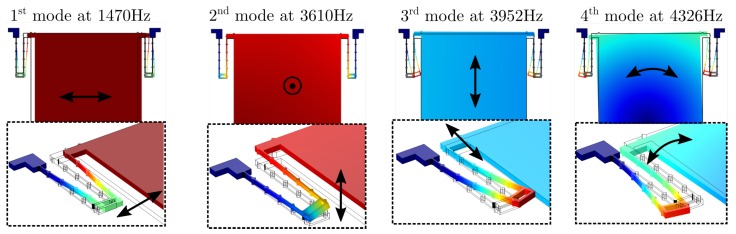
Results of four significant eigenfrequencies obtained by FEM simulation. A single mass with neglected holes and a Young’s modulus of $E_{Y}=130\;\mathrm{GPa}$ (corresponding to Si <100>) was investigated. The $1^{\mathrm{st}}$ eigenmode is of interest, referring to in-plane vibration at $f_{r}=$ 1470 Hz.

A Lorentz force driven mechanical system can be described by a harmonic oscillator with a steady-state oscillation amplitude of

(1) $$A\left(\omega \right)=\frac{L_{e}\;i_{\mathrm{ac}}\;B}{m\sqrt{{\left(\omega_{0}^{2}-\omega^{2}\right)}^{2}+{\left(2\zeta \omega \omega_{0}\right)}^{2}}},$$

where $L_{e}\;i_{\mathrm{ac}}\;B$ denotes the force acting on the resonator’s body, *m* denotes the body’s mass and ω the angular frequency of the excitation force. In the regime of linear elastic material behaviour, i.e., small spring deflection, the natural frequency $\omega_{0}=\sqrt{k/m}$ and damping ratio $\zeta =c/(2\sqrt{km})$ can be modelled by a lumped parameter approach with stiffness *k* and damping coefficient *c*. To estimate the masses distinct mode of vibration, a finite element method (FEM) simulation was performed (Figure 3).

The second mode occurs at 2.5$f_{r}$ factor roughly, suggesting a negligible contribution when excited at $f_{r}$. Equation (1) describes a linear dependency between the body’s deflection and the applied magnetic field. Transferring this principle to the presented sensor comprising two identical masses, the perpendicular component of the magnetic flux density can be simultaneously measured at two points in space. Hereby, the spatial error is only given by the micromachining process which favours sub-micrometer resolution. From the measured magnetic flux densities $B_{x,\;\mathrm{mL}}$ and $B_{x,\;\mathrm{mR}}$ occurring at the masses $m_{L}$ and $m_{R}$, respectively, the magnetic field gradient at one point in space is estimated with the partial derivative

(2) $$G_{y}=\frac{\partial B_{x}}{\partial y}\approx \frac{B_{x,\;\mathrm{mL}}-B_{x,\;\mathrm{mR}}}{d_{\mathrm{sens}}},$$

where $d_{\mathrm{sens}}$ denotes the fixed distance of the masses’ sensing edges. Concerning the designed offset $d_{\mathrm{off}}$ between stationary Cr pattern and Si holes, the field direction is determined by measuring the phase signal. If a gradient field is present (Case 1), i.e., the perpendicular component of the field direction reverses at the center of the transducer, the masses deflect in opposite direction. Hence, the apertures either close or open simultaneously, resulting in a zero phase difference. In contrast, a unidirectional *B*-field induces deflection in the same direction. One mass will open the apertures whereas the other mass will close them, resulting in a phase difference of $\varphi_{\mathrm{diff}}=\pm 180$. Thus, only the masses oscillation magnitude denotes either a constant field (Case 2) or constant field superimposed with a gradient field (Case 3) when the magnitudes are equal or differ, respectively. The direction of the measured magnetic flux density according to the phase signal can be summarised as

(3) φmLφmRBx,mLBx,mR180∘0∘↑↑180∘180∘↑↓0∘180∘↓↓0∘0∘↓↑φdiff=φmL−φmR=−180∘→Bx(Case2or3)0∘→Gy(Case1)180∘→−Bx(Case2or3),

where the arrows denotes the perpendicular field component either pointing to (↑) or away (↓) from the foreside of the mass.

## 3. Sensor Characterisation & Measurements

The MEMS sensor was characterised in two ways and its performance demonstrated by mapping a bar magnet’s *B*-field and gradient in 3D-space. First, the mechanical properties were investigated by recording the frequency response for a constant applied magnetic field. From this approach, the sensor’s resonant frequency $f_{r}$ and quality factor Q=1/(2ζ) were extracted. Further, the behaviour of the phase signal was recorded to distinguish the field direction and, hence, the possible cases (1–3) as discussed in Section 2. Second, the sensor was investigated within a homogeneous magnetic field generated by coils in Helmholtz configuration and afterwards within a (pure) gradient field (Case 1) produced by antisymmetric placement of neodymium (Nd) magnets. Here, the sensor was driven at $f_{r}$ and only the field strength was changed to extract the masses’ response $S=\partial U_{\mathrm{out}}/\partial B$ and detection limits DT.

**Figure 4:**
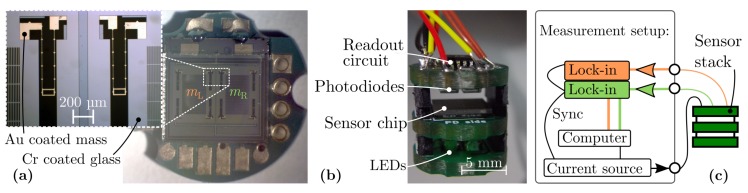
(**a**), Sensor chip bonded onto a PCB board, depicting the two masses $m_{L}$ and $m_{R}$. (**b**), Assembled stack including a readout circuit (transimpedance amplifier), photodiodes, sensor chip and LEDs. (**c**), Schematic of the measurement setup.

The Si chip as a ‘bare die’ bonded onto a printed circuit board (PCB) and the fully assembled sensor device assembled as stack is depicted in Figure 4.

The on-board electronic of the stack includes LED driver and two transimpedance amplifiers to convert the photocurrents through the photodiodes into an amplified output voltage. A current source (model Keithley 6221) provided the excitation current $i_{\mathrm{ac}}$ and the detected light flux was acquired with lock-in amplifiers (Stanford Research Systems SR830 and SR865). A computer controlled the measurement setup at all time (Figure 4c). All forthcoming measurements were conducted at ambient air pressure and an excitation current of 800 µA.

### 3.1. Frequency Response

**Figure 5:**
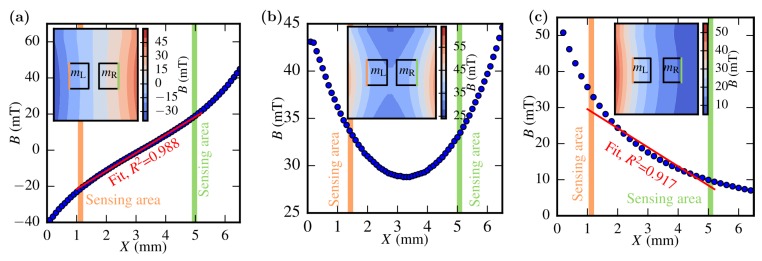
Results of the measured B−field distribution for different magnet settings characterised with a hall probe (Teslameter FM302, AS-HAP, Project Elektronik). The insets show the estimated position of the masses within the field with the sensing area located at the outermost edges, highlighted orange and green for $m_{L}$ and $m_{R}$, respectively. (**a**) Anti-symmetric placement of the magnets yields a pure gradient field (Case 1). (**b**) Homogeneous magnetic field by symmetric placement of the magnets (Case 2). (**c**) A gradient field with offset occurs, if only one magnet is used (Case 3).

**Figure 6:**
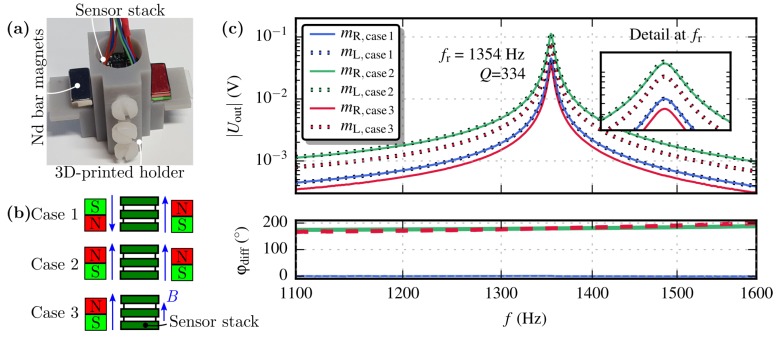
(**a**), Measurement setup to characterise the sensor’s frequency response. (**b**), Schematic of different magnet configuration.(**c**), Response of the MEMS gradiometer for the three different magnet settings in Figure 5. The measured transfer functions of both masses are shown and a quality factor of Q=334 was extracted via the −3 dB bandwidth method. The corresponding phase difference $\varphi_{\mathrm{diff}}=\varphi_{m_{L}}-\varphi_{m_{R}}$ is plotted at the bottom.

The frequency response was recorded by placing the sensor between two Nd magnets. Depending on the magnet configuration, a gradient field (Case 1), homogeneous field (Case 2) and offset-gradient field (Case 3) was generated (see Figure 5 and Figure 6).

The field distribution was first measured with an axial hall probe serving as reference for the sensor. From this, the actual position of the masses and, hence, the sensing edges have been determined. A gradient field of 10 mT/mm was calculated from the linearised fit-function for anti-symmetric placements of the magnets (Figure 5a). Figure 5b depicts the field distribution occurring from symmetric placements of the magnets. A small difference in the magnetic field of 160 µT was found when determining the magnetic flux densities at the sensing areas. A single magnet, placed on the left side of the 3D-printed holder generates a gradient field of 5.3 mT/mm with offset in the vicinity of the sensor’s masses (Figure 5c). The measured transfer function for all three cases and measurement setup is shown in Figure 6.

The masses oscillate in opposite direction when a gradient field (Case 1) is present. Hence, the apertures either close or open simultaneously, thus the difference of the phase $\varphi_{\mathrm{diff}}$ is zero. Ideally, the masses detect the same magnetic field by symmetric placement of the identical magnets (Case 2). Nevertheless, the output signals insignificantly differs as depicted in the detail view of the recorded transfer functions. This nonconformity originates from 3D-printing tolerances of the holder, placement errors of the Si chip onto the PCB board and differences at the magnets’ strengths. However, $\varphi_{\mathrm{diff}}=$ 180${}^{\circ }$ was obtained indicating that both masses deflect in the same direction. A single magnet generates a gradient field with offset (Case 3) which is measured by the MEMS sensor. Here, $\varphi_{\mathrm{diff}}$ is also 180${}^{\circ }$ and the difference of the masses’ magnitude solely indicates the gradient.

The measured resonance frequency $f_{r}=1354$ Hz is close to the result from the eigenfrequency obtained by FEM simulation (deviates about 8.6%). Furthermore, a negligible deviation of 0.6 Hz between both masses’ resonance peak was observed.

### 3.2. Magnitude Response

**Figure 7:**
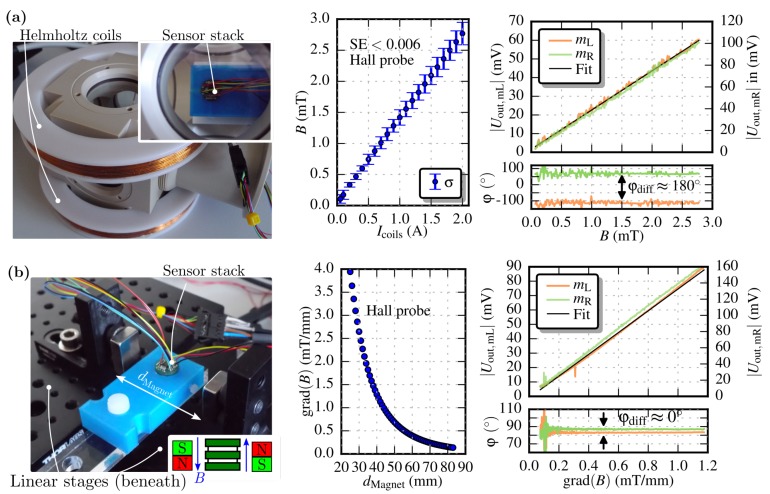
(**a**), Helmholtz configuration to characterise the sensor within a homogeneous magnetic field, which was calibrated with a hall probe (Teslameter, AS-NTM-2). A standard error of SE <0.006 was obtained from the calibration measurement within an area of 20 mm${}^{2}$, which affirms the homogeneity of the field. The masses response is shown on the right. (**b**), Gradient field setup. The sensor stack is centered between two Nd magnets which are fixed onto linear stages. The distance $d_{\mathrm{Magnet}}$ is gradually changed to generate different gradients fields. Again, the field was measured by a hall probe and gradient calculated prior to monitoring the masses’ response.

Referring to Equation (1), the magnitudes of the Lorentz force-excited masses depend linearly on the magnetic field. The masses were excited at their resonance frequency and the external field was varied to obtain the sensor’s responsivity *S*. Figure 7a depicts the sensor’s output signals with respect to a homogeneous magnetic field generated by coils in Helmholtz configuration (Case 2). The occurring field created by the coils was first measured by a Hall probe within an area of 20 mm${}^{2}$ for different current settings. The uniformity of the magnetic field at this area is affirmed with an obtained standard error of SE <0.006. A responsivity of $S_{fr,\;mL}=19.04$ V/T and $S_{fr,\;mR}=35.65$ V/T for $m_{L}$ and $m_{R}$, respectively, was extracted from linear fit functions (coefficient of determination $R^{2}=0.997$). A phase difference of 180${}^{\circ }$ indicates that both masses oscillates in the same direction which affirms the presence of a unidirectional field. Note, that the output signals deviate by a factor, $U_{\mathrm{out},\;m_{L}}\approx 1.87\;U_{\mathrm{out},\;m_{R}}$. This might be caused due to fabrication tolerances and variations of the width of the current conducting paths on the masses. The latter may occur from a small offset of the mask during the photolithography step. Emerging deviation of the leads’ conductivity across the masses results in an asymmetric partition of the current and, thus, diverging magnitudes of mass deflection in a homogeneous magnetic field.

The sensor was also investigated with different varying gradient fields that occur between Nd magnets (Case 1, see Figure 7b) mounted on motorised linear stages. Different gradient fields were generated by changing the distance $d_{\mathrm{Magnet}}$ between the magnets. The occurring field between both magnets was first measured with a hall probe along an 8 mm line. Further, the gradient field was extracted and the location of the masses’ sensing ledges identified within the field. These measured gradient values serve as basis to perform the measurements with the MEMS sensor. The measured responses to a change of the gradient field were 77.57 V/(T/mm) and 139.12 V/(T/mm) for $m_{L}$ and $m_{R}$, respectively. From this, the field responsivities are calculated to be $S_{fr,\;mL}=19.89$ V/T and $S_{fr,\;mR}=35.67$ V/T with the distance $d_{\mathrm{sens}}=3.9\;\mathrm{mm}$ between the masses’ sensing ledge. These values agree very well with the sensitivities obtained from the Helmholtz setup. Again, the output signals deviate about a factor of ≈1.8. This value marginally differs from the factor obtained from the measurements with the Helmholtz configuration (≈1.87). These errors are due to the non-ideal alignment of the magnets and the chip-holder, which causes additional higher order inhomogeneities. Further, the measured phase difference is almost zero, indicating that both masses move in opposite directions, and, hence, a (pure) gradient field. The small deviation of $\varphi_{\mathrm{diff}}\leq 15^{\circ }$ occurs from the unwrap mechanism of the phase signal. The phase signal also indicates the beginning of the noise range at field gradients <0.1 mT/mm.

The sensor’s noise floor was measured to be 1 mV at resonance, leading to a detection limit of $DT_{mL}=5.75$ µT/$\sqrt{\mathrm{Hz}}$ and $DT_{mR}=3.07$ µT/$\sqrt{\mathrm{Hz}}$, taking into account the equivalent noise bandwidth ENBW of 83.3 Hz of the lock-in amplifiers.

### 3.3. 3D Magnetic Field Characterisation of a Nd Bar Magnet

**Figure 8:**
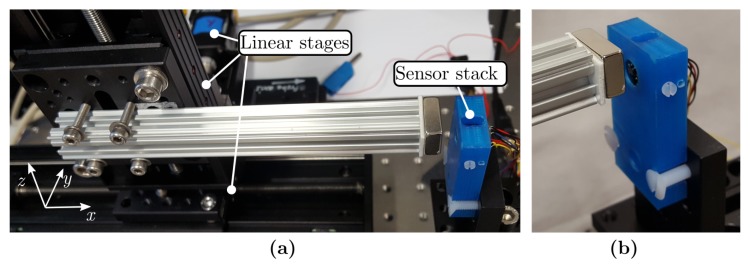
(**a**), Setup for characterisation of the magnetic flux density and gradient field of the Nd bar magnet. The magnet was mounted onto an aluminium shaft fixed to three linear stages (PLS-85, Micos SMC Corvus ECO Positioning Controller) to enable scanning of the magnetic field. (**b**), The sensor stack is mounted in a plastic housing and fixed on a breadboard.

In order to demonstrate the sensor’s performance, the distribution of the magnetic flux and gradient field in proximity of a Nd bar magnet was measured and compared with simulation results. The magnet with size of 19×12.7×6.35 mm${}^{3}$ and remanent magnetisation of 1.3 T was mounted onto a non-magnetic aluminium shaft connected to three motorised linear stages (Figure 8).

**Figure 9:**
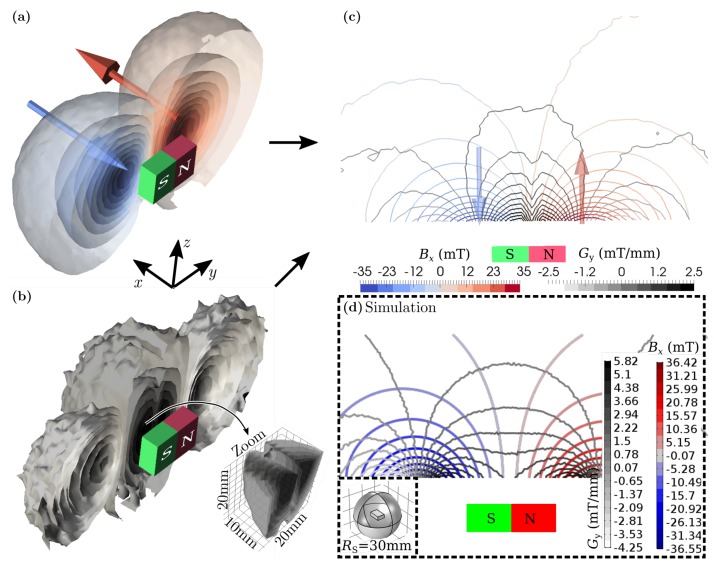
Mapping of the bar magnet’s magnetic flux density $B_{x}$ (**a**) and derived gradient field $G_{y}$ (**b**). The xy-plane cut through the magnet’s center shows both, $B_{x}$ and $G_{y}$ combined (**c**). In comparison, simulation results of magnetic flux density and gradient field of a bar magnet is shown in (**d**). Note, only the *x*-component of the field is represented.

In order to obtain quantitative results, the aforementioned calibration curves (see Section 3.2) are used. A volume of ≈105,000 mm${}^{3}$ with 2 mm step-size was mapped, corresponding to a total of 32,500 measured points. The results were interpolated and graphically represented (Figure 9). Based on a xy-plane cut through the magnet’s center, the measurement was compared with simulation data of an ideal bar magnet of same size and an assumed magnetisation of 500 kA/m. The simulated and measured magnetic flux density and gradient field are qualitatively consistent. High gradient field values occur very close to the magnet where a change of the magnetic field is large.

## 4. Conclusions and Outlook

The theoretical detection limit of resonators is related to the thermo-mechanical noise [23]. The mean noise force is expressed as $F_{n}=\sqrt{4k_{B}T\;c}$, where $k_{B}$, *T* and *c* are the Boltzmann constant, the temperature and damping coefficient, respectively. Hence, the minimum detectable magnetic flux density amounts to $B_{\min}=\sqrt{4k_{B}T\;c}/\left(i_{\mathrm{ac}}L_{e}\right)=126\;\mathrm{nT}/\sqrt{\mathrm{Hz}}$, taking into account the estimated damping coefficient from the measurement. However, the performance of the sensor differs roughly by a factor of 45 (see the detection limit DT in Table 2). This limit is mainly determined by the electronic noise of the readout. Hence, a more finely tuned amplifier circuitry may increase the detection limit. Another approach is to reduce the damping factor, e.g., by operating the resonator in vacuum. This could be realised by sealing the backside of the Si chip with translucent glass so as not interfere with the optical readout.

In Section 3.2, it was shown that the masses’ responsivities deviate roughly by a factor of 1.8. Apparently, the excitation current is not equally distributed via the masses and, hence, resulting in a different mass deflection magnitude. The fact, that both masses have the same resonance frequency and quality factor, i.e., the same damping behaviour, supports the assumption of the asymmetric distribution of the current. This effect should be further investigated by considering two separated current paths, one for each mass, in a future sensor design. Additionally, independently excitable masses enables simultaneous measurement of the magnetic field and ambient vibrations on a single chip. Thus, one mass detects an external *B*-field whereas the other mass measures the vibration. Consequently, additional information about ambient noise may enhance the sensor’s detection limit.

In summary, the proposed dual-resonator MEMS gradiometer is capable of detecting and differentiating between gradient fields (Case 1), homogeneous fields (Case 2) and gradient fields superimposed with a homogeneous field (Case 3) by measuring the magnetic flux density simultaneously at two points in space. The simplified local evaluation of the field gradient due to the known distance of the masses is promising for an accurate characterisation of magnetic fields. This also eliminates positioning errors compared to a step-by-step measurement with a commercially available single hall probe and subsequently calculation of the gradient. Furthermore, the sensor is scalable by adding additional two masses, rotated by 90${}^{\circ }$. This would enable the detection of a second gradient component on a single Si chip, manufactured with the same micromachining processes.

## Figures and Tables

**Table 1:** Summary of various Lorentz force-based resonant MEMS magnetic-field sensors.

Publication	Sensing	Resonant	Quality	Detection	Dimension
	Technique	Frequency	Factor	Limit	
		kHz		nT/$\sqrt{\mathrm{Hz}}$	mm × mm
Kumar et al. [8]	Piezoresistive	400	1.1 × $10^{6}$	2.76 × $10^{-3}$	0.8 × 0.8
				@7.245 mA	(single mass)
Park et al. [9]	Optical	0.364	116	11.6	3.3 × 3.3
				@50 mA	
Sonmezoglu et al. [5]	Capacitive	47.22	11760	50	2.2 × 2.2
				@1.1 mA	(die)
Laghi et al. [10]	Capacitive	19.34	790	185	1.6 × 0.85
				@100 µA	(single device)
Herrera-May et al. [11]	Piezoresistive	100.7	419.6	2500$\sqrt{\mathrm{Hz}}$	0.472 × 0.3
				@10 mA	(mass only)

**Table:** 

*A*	*t*	$L_{e}$	*m*	$f_{r}$	*Q*	*k*	*c*
${\mathrm{mm}}^{2}$	µm	mm	$\times 10^{-9}\;\mathrm{kg}$	Hz		N/m	µg/s
4	45	2.5	38.25	1354	334	2.77	0.974

**Table:** 

$S_{fr,\;mL}$	$DT_{mL}$	$S_{fr,\;mR}$	$DT_{mR}$
V/T	µT/$\sqrt{\mathrm{Hz}}$	V/T	µT/$\sqrt{\mathrm{Hz}}$
19.04	5.75	35.65	3.07
